# Clinical Relevance and Environmental Prevalence of *Mycobacterium fortuitum* Group Members. Comment on Mugetti et al. Gene Sequencing and Phylogenetic Analysis: Powerful Tools for an Improved Diagnosis of Fish Mycobacteriosis Caused by *Mycobacterium fortuitum* Group Members. *Microorganisms* 2021, *9*, 797

**DOI:** 10.3390/microorganisms9112345

**Published:** 2021-11-12

**Authors:** Ivo Pavlik, Vit Ulmann, Ross Tim Weston

**Affiliations:** 1 Faculty of Regional Development and International Studies, Mendel University in Brno, Tr. Generala Piky 7, 613 00 Brno, Czech Republic; 2 Public Health Institute Ostrava, Partyzanske Nam. 7, 702 00 Ostrava, Czech Republic; vit.ulmann@zuova.cz; 3 Department of Biochemistry and Genetics, La Trobe Institute for Molecular Science, La Trobe University, Bundoora, Melbourne, VIC 3086, Australia; R.Weston@latrobe.edu.au

**Keywords:** non-tuberculous mycobacteria, saprophytic environmental mycobacteria, potentially pathogenic mycobacteria, fish directed for consumption, environmental prevalence

## Abstract

*Mycobacterium fortuitum* group (MFG) members are able to cause clinical mycobacteriosis in fish and other animals including humans. *M. alvei*, *M. arceuilense*, *M. brisbanense*, *M. conceptionense*, *M. fortuitum*, *M. peregrinum*, *M. porcinum*, *M. senegalense, M. septicum*, and *M. setense* were isolated from fish with mycobacteriosis. In other animals only three MFG species have been isolated: *M. arceuilense* from camels’ milk, *M. farcinogenes* from cutaneous infections often described as “farcy”, and *M. fortuitum* from different domestic and wild mammals’ species. Out of 17, only 3 MFG species (*M. arceuilense*, *M. lutetiense* and *M. montmartrense*) have never been reported in humans. A total of eight MFG members (*M. alvei*, *M. brisbanense*, *M. conceptionense*, *M. fortuitum* subsp. *acetamidolyticum*, *M. houstonense*, *M. peregrinum*, *M. porcinum*, and *M. septicum*) have been isolated from both pulmonary and extrathoracic locations. In extrathoracic tissues five MFG species (*M. boenickei*, *M. farcinogenes*, *M. neworleansense*, *M. senegalense*, and *M. setense*) have been diagnosed and only one MFG member (*M. fortuitum* subsp. *acetamidolyticum*) has been isolated from pulmonary infection.

We would like to comment on a recent article published in your journal by Mugetti et al. [1]. This article is well written and discusses the diagnostics of all 17 members of the *Mycobacterium fortuitum* Group (MFG) currently known. The aim of the study was the improvement of species diagnostics with respect to pathogenicity of all MFG members in 130 freshwater and saltwater fish with pathognomonic pathological lesions in parenchymatous organs and the skin. The field is divided by the fact that while many publications include most MFG members as fish pathogens, another significant number of publications describe these MFG members only as saprophytic mycobacteria present in the water environment (biofilm, sediment, water plants, non-vertebrates and other matrices) or on healthy animals including fish. To this end we have summarised published results concerning the prevalence of MFG members in the environment and their clinical relevance to humans and animals (including fish). Specifically, we question whether these MFG members are not only pathogenic/virulent to fish, but also to other animals and humans from an epidemiological and epizootiological point of view.

## 1. Clinical Relevance in Fish

*Mycobacterium fortuitum* was first described in 1938 [2] and since that time, during last two decades in particular, the remaining MFG members have been identified (Table 1). A total of 11 MFG members have been isolated from fish. Prior to a study by Mugetti et al. [1], only *M. fortuitum*, *M. peregrinum*, *M. porcinum*, *M. septicum* and *M. setense* were described as fish pathogens. In their study, Mugetti et al. [1] described for the first time five MFG members (*M. alvei*, *M. arceuilense*, *M. brisbanense*, *M. conceptionense* and *M. senegalense*) as species isolated from fish with mycobacteriosis (Table 1). These five MFG species were isolated from 10 genera of freshwater fish originally living in tropical and temperate zones. *M. alvei* and *M. brisbanense* were detected in one fish genus each. Three MFG species were isolated from fish of three (*M. arceuilense*), four (*M. conceptionense*) and six (*M. senegalense*) genera (Table 2). This finding could indicate that these MFG members could be considered as emerging fish pathogens.

Three MFG species (*M. conceptionense*, *M. houstonense* and *M. senegalense*) were previously isolated only rarely from fish directed for consumption, without known clinical relevance for the fish [7]. In the study of Mugetti et al. the pathogenicity for fish was documented in two of these MFG species: *M. conceptionense* and *M. senegalense* [1]. Two MFG species (*M. fortuitum* and *M. peregrinum*) in this study were diagnosed in 10 and 13 fish genera, respectively (Table 2); however, the relevance of this observation is questionable. This high prevalence in fish with mycobacteriosis was more frequently diagnosed in significantly earlier studies, as shown in Table 1, when diagnostics were not as sophisticated as they are now [1] and it is possible that misidentification of these species has occurred in some of these cases.

## 2. Clinical Relevance in Other Animals

Surprisingly in other animals (specifically mammal, not including humans), only three MFG species have been isolated: *M. arceuilense* from camels’ milk, *M. farcinogenes* from cutaneous infections often described as “farcy” and *M. fortuitum* from different domestic and wild mammal species (Table 1). These relatively rare cases are in contrast with MFG species detection in humans, described below.

## 3. Clinical Relevance in Humans

Out of 17, only 3 MFG species (*M. arceuilense*, *M. lutetiense* and *M. montmartrense*) have not ever been reported in humans. A total of eight MFG members (*M. alvei*, *M. brisbanense*, *M. conceptionense*, *M. fortuitum* subsp. *acetamidolyticum*, *M. houstonense*, *M. peregrinum*, *M. porcinum*, and *M. septicum*) have been isolated from both pulmonary and extrathoracic tissues. In extrathoracic tissues, five MFG species (*M. boenickei*, *M. farcinogenes*, *M. neworleansense*, *M. senegalense*, and *M. setense*) have been diagnosed and only one MFG member (*M. fortuitum* subsp. *acetamidolyticum*) has been isolated in pulmonary infection. Three MFG species (*M. arceuilense*, *M. lutetiense*, and *M. montmartrense*) have not ever been reported in humans (Table 1).

The fact that most MFG species have been isolated in humans, and more rarely in animals and the environment, is likely due to a lack of research. The source of mycobacteriosis caused by MFG member infections reported in humans is often suspected to be from the immediate environment, such as living spaces, hospitals, gardens, swimming pools, surface waters, etc. [31]; however, few research groups actively demonstrate that the environment is the source. Only a study by Konjek et al. [4], focused on the water distribution system in which the presence of *M. lutetiense* and *M. montmartrense* was described and research done by Davarpanah et al. [30] studying the presence of MFG members in hospital soil and dust in Iran are exceptions to this. This work by Mugetti et al. [1] should encourage further investigation into environmental sources of MFG infections. Further research should also be conducted to evaluate the clinical relevance of remaining MFG species previously not reported in humans.

## 4. Environmental Prevalence

The ecology of mycobacteria, including all known MFG species (up until 2009) was described by Kazda et al. [31]. At that time 14 of 17 MFG members had been described previously (Table 1). Over the subsequent decade we have continued the study of mycobacterial ecology concerning MFG members. We have described 12 MFG members isolated from the environment and non-vertebrates, including from peat [32,33], soil and earthworms [34], aquariums and fishponds [35], prawns used for fish feed and aquarium plants such as *Vesicularia dubyana* and *Cryptocoryne wendtii* [36], alluvial wooden material in karstic caves [37] and in other matrices (unpublished data). Only five MFG members (*M. boenickei*, *M. farcinogenes*, *M. fortuitum* subsp. *acetamidolyticum*, *M. houstonense*, and *M. neworleansense*) have not ever been reported in the environment (Table 1). From an epidemiological and epizootiological point of view, we suspect that the environmental presence of MFG members plays an important role in the spreading and host exposure of MFG members.

## 5. Conclusions

**Figure 1:**
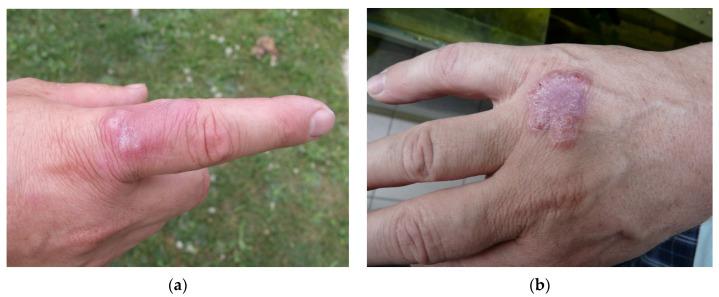
Fish tank granuloma diagnosed in professional aquarist on two places on his right hand: (**a**) infection on 17 June 2003; (**b**) infection on 2 July 2008 (photo I. Pavlik).

In the publication of Mugetti et al., the current taxonomy of MFG members is shown and an improvement to the methods for identifying clinically relevant and environmental isolates is demonstrated [1]. As well as fish, MFG members are able to cause clinical mycobacteriosis in other animals including humans. Therefore, the detection of new MFG species in fish could indicates new risks for humans, in particular for fish handlers, aquarists and their family members (Figure 1). MFG species have often been diagnosed in human skin infections in a condition previously known as “swimming pool granuloma”. A decrease in the prevalence of this condition is thought to be due to improvements in swimming pool management. Consequently, this condition is mostly seen in infected people associated with aquarium fish, and currently skin mycobacteriosis is most often described as “fish tank granuloma” [31]. Results presented by Mugetti et al. open new perspectives in the rapid identification of MFG members not only in fish, but also in other hosts and the environment [1]. We envisage that this added information concerning the clinical relevance of MFG members and their environmental prevalence will significantly extend the knowledge of this field.

## Figures and Tables

**Table 1:** *Mycobacterium fortuitum* Group members’ characteristics (year of description, pathogenicity/virulence for humans and animals, environmental prevalence and detection in fish).

Member of	Year **	Clinical Significance			Detection in	Isolates ***
MFG *		Humans	Animals	Fish	Environment	No. (%)
*M. alvei*	1992	Rare pulmonary and extrathoracic pathogen [3]	Never reported	For the first time reported in infected fish [1]	Reported [4]	2 (1.5)
*M. arceuilense*	2016	Never reported	Camels’ milk isolates [5]	For the first time reported in infected fish [1]	Reported [4]	4 (3.1)
*M. boenickei*	2004	Rare extrathoracic pathogen [3]	Never reported	Never reported	Never reported	0
*M. brisbanense*	2004	Rare pulmonary and extrathoracic pathogen [3]	Never reported	For the first time reported in infected fish [1]	Reported [6]	4 (3.1)
*M. conceptionense*	2006	Rare pulmonary and extrathoracic pathogen [3]	Never reported	Fish directed for consumption [7], for the first time reported in infected fish [1]	Reported [8]	4 (3.1)
*M. farcinogenes*	1973	Rare extrathoracic pathogen [9]	Cutaneous infection “farcy” [10,11,12]	Never reported	Never reported	0
*M. fortuitum ssp. acetamidolyticum*	1986	Rare pulmonary pathogen [13]	Never reported	Never reported	Never reported	0
*M. fortuitum*	1938 (1986)	Rare pulmonary and extrathoracic pathogen [14,15,16,17]	Domestic and wild mammal pathogen [18,19,20,21,22]	Fish pathogen [1,23]	Reported [24]	38 (29.2)
*M. houstonense*	2004	Rare pulmonary and extrathoracic pathogen [3]	Never reported	Fish directed for consumption [7]	Never reported	0
*M. lutetiense*	2016	Never reported	Never reported	Never reported	Reported [4]	0
*M. montmartrense*	2016	Never reported	Never reported	Never reported	Reported [4]	0
*M. neworleansense*	2004	Rare extrathoracic pathogen [25]	Never reported	Never reported	Never reported	0
*M. peregrinum*	1962 (1992)	Rare pulmonary and extrathoracic pathogen [26,27]	Never reported	Fish pathogen [1,28]	Reported [24]	63 (48.5)
*M. porcinum*	1983	Rare pulmonary and extrathoracic pathogen [3]	Never reported	Fish pathogen [28]	Reported [8]	0
*M. senegalense*	1973 (1980)	Rare extrathoracic pathogen [3,23]	Never reported	Fish directed for consumption [7], For the first time reported in infected fish [1]	Reported [24]	12 (9.2)
*M. septicum*	2000	Rare pulmonary and extrathoracic pathogen [3]	Never reported	Fish pathogen [1,28,29]	Reported [24]	1 (0.8)
*M. setense*	2008	Rare extrathoracic pathogen [3]	Never reported	Fish pathogen [1,29]	Reported [30]	2 (1.5)
Total						130 (100)

Table interpretation. * In alphabetical order; ** Year of description [2] (LPSN: List of Prokaryotic names with Standing in Nomenclature https://lpsn.dsmz.de/ (accessed on 30 September 2021); *** Mugetti et al. [1] (Table 2).

**Table 2:** Characteristics of examined infected freshwater and saltwater fish with mycobacteriosis in the study carried out by Mugetti et al. [1].

Species	Species	Continent	Water	Climate	MFG
(Latin) *	(English)	(Origin) **	Type	Zone	Member
*Acipenser ruthenus*	Sterlet	AF	FW	TeZ	*M. peregrinum*
*Astatotilapia obliquidens*	Zebra *Obliquidens*	AF (Uganda)	FW	TrZ	*M. fortuitum*
*Aulonocara* sp.	Aulonocara Fire Fish	AF (Malawi)	FW	TrZ	*M. fortuitum, M. peregrinum*
*Botia macracantha*	Clown Loach	AS	FW	TrZ	*M. senegalense*
*Capoeta tetrazona*	Partbelt Barb	AS	FW	TrZ	*M. senegalense*
*Carassius auratus*	Goldfish	AS	FW	TeZ	*M. alvei, M. arceuilense, M. conceptionense, M. fortuitum, M. peregrinum, M. senegalense, M. septicum*
*Colisa lalia*	Dwarf Gourami	AS	FW	TrZ	*M. conceptionense, M. peregrinum*
*Copadichromis borleyi*	Haplochromis Borleyi Redfin	AF (Malawi)	FW	TrZ	*M. fortuitum, M. peregrinum*
*Copadichromis* sp.	Copadichromis	AF (Malawi)	FW	TrZ	*M. fortuitum, M. peregrinum*
*Cyprinus carpio* var. *koi*	Koi	AS	FW	TeZ	*M. arceuilense, M. peregrinum, M. senegalense*
*Dicentrarchus labrax*	Common Bass	AO and MS	SW	Sea ***	*M. fortuitum*
*Garra rufa*	Doctor Fish	AS	FW	TrZ	*M. fortuitum, M. peregrinum, M. setense*
*Hypostomus plecostomus*	Plecostomus	AM (South)	FW	TrZ	*M. peregrinum*
*Maylandia lombardoi*	Lombardoi Mbuna	AF (Malawi)	FW	TrZ	*M. peregrinum*
*Misgurnus* sp.	Loach	Eurasia	FW	TeZ	*M. peregrinum*
*Nimbochromis livingstonii*	Livingston’s Cichlid	AF (Malawi)	FW	TrZ	*M. fortuitum, M. peregrinum*
*Nimbochromis venustus*	Giraffe Hap	AF (Malawi)	FW	TrZ	*M. fortuitum, M. peregrinum*
*Placidochromis* sp.	Placidochromis	AF (Malawi)	FW	TrZ	*M. fortuitum, M. peregrinum, M. senegalense*
*Poecilia latipinna*	Sailfin Molly	AM (Central)	FW	TrZ	*M. arceuilense, M. fortuitum*
*Poecilia reticulata*	Barbados Millions	AM (South)	FW	TrZ	*M. fortuitum*
*Pseudotropheus* sp.	Mbuna Cichlid	AF (Malawi)	FW	TrZ	*M. peregrinum*
*Pterophyllum scalare*	Black Angelfish	AM (South)	FW	TrZ	*M. conceptionense*
*Sciaenops ocellatus*	Channel Bass	AF (Tanganyika)	FW	TrZ	*M. brisbanense*
*Symphysodon discus*	Discus	AM (South)	FW	TrZ	*M. conceptionense, M. fortuitum, M. senegalense*
*Xiphophorus maculatus*	Moon Fish	AM (Central)	FW	TrZ	*M. peregrinum*

Table interpretation. * In alphabetical order; ** Original occurrence of the fish species; *** Sea of temperate and subtropical belt; AF = Africa; AS = Asia; AO and MS = Atlantic Ocean and Mediterranean Sea; AM = America; MFG = *Mycobacterium fortuitum* Group; FW = Freshwater; SW = Saltwater; TeZ = Temperate Zone; TrZ = Tropical Zone; *M.* = *Mycobacterium*.

## Data Availability

Availability of data and materials correspondence be addressed to the corresponding author.
